# Gastrointestinal Complications of COVID-19 Vaccines

**DOI:** 10.7759/cureus.24070

**Published:** 2022-04-12

**Authors:** Kunal Ajmera, Rashika Bansal, Heather Wilkinson, Lokesh Goyal

**Affiliations:** 1 Hospital Medicine, Calvert Health Medical Center, Prince Frederick, USA; 2 Endocrinology, Diabetes and Metabolism, Cleveland Clinic, Cleveland, USA; 3 Critical Care Medicine, Doctor’s Community Hospital, Lanham, USA; 4 Hospital Medicine, Christus Spohn, Corpus Christi, USA

**Keywords:** diverticulitis colon, gastrointestinal complications., adverse events, covid-19 vaccine, covid-19

## Abstract

Much of the control over the coronavirus disease 2019 (COVID-19) pandemic has been achieved by mass vaccination against the severe acute respiratory syndrome coronavirus 2 (SARS-CoV-2), the etiologic agent that causes COVID-19. The COVID-19 mRNA (messenger RNA) vaccines are relatively newly approved and have been widely used in the US since they first became available. However, with passing time, data regarding adverse events associated with the mRNA vaccines have become clearer. Vaccines are safe in general, and the benefits outweigh the risks of adverse events. In this case report, we present the first documented case report of post-vaccination acute diverticulitis and colon micro-perforation following Moderna booster dose (Moderna Inc, Cambridge, USA) in a young adult. Vaccine recipients should be educated on vaccine-associated gastrointestinal (GI) adverse events in order to reduce morbidity and mortality. We also recommend that vaccine recipients with pre-existing GI disorders should be carefully monitored for the worsening of pre-existing conditions post-COVID-19 vaccination.

## Introduction

The first case of coronavirus disease 2019 (COVID-19), caused by the novel severe acute respiratory syndrome-coronavirus 2 (SARS-CoV-2), was diagnosed in December 2019 in Wuhan, China. The disease has since rapidly spread all over the world, leading to the declaration of COVID-19 as a pandemic by the World Health Organization on March 11, 2020. Since the first appearance of SARS-CoV-2, billions of people worldwide have been diagnosed as infected, and millions have died worldwide from this disease as of March 4, 2022 [1]. As per US Centers for Disease Control and Prevention (CDC) data, almost 80 million cases and 1 million deaths have been reported in the US alone [2]. Since the beginning of the pandemic, different preventative and therapeutic approaches have been adopted and implemented globally to control the pandemic. Vaccines have been used as a primary disease prevention measure for centuries, and their use has helped in effectively curbing infection rates in the current pandemic. The widely available vaccines in the US include two mRNA (messenger RNA) vaccines, Pfizer-BioNTech (BNT162b2; Pfizer Inc, New York City, USA and BioNTech SE, Mainz, Germany) and Moderna (mRNA-1273; Moderna Inc, Cambridge, USA), and the Janssen vaccine made of modified adenovirus DNA (Johnson & Johnson, New Brunswick, USA). [3,4]

Diverticulosis is the outpouchings of the bowel wall, occurring most commonly in the sigmoid colon in Western countries, whereas diverticulitis is inflammation of the diverticula of the bowel wall. Over 50% of people over age 60 and over 60% of people over age 80 have colonic diverticula. [5] The lifetime risk of developing diverticulitis is estimated at 5-25% [6-8]. Diverticulitis, on average, accounts for an estimated 371,000 emergency department visits and 200,000 inpatient admissions per year, costing 2.1-2.6 billion dollars per year in the United States [9]. Other risk factors for diverticular disease include advancing age, use of non-steroidal anti-inflammatory drugs (NSAIDs) (including aspirin), steroids, and opioids, along with smoking and a sedentary lifestyle.

The clinical presentation for acute diverticulitis may vary, but left lower quadrant abdominal pain is the most common symptom. Other non-specific symptoms include fever and malaise. The gold standard diagnostic test includes a CT scan of the abdomen, with sensitivity and specificity of over 98%. Here, we present the first published case report of post-vaccination acute diverticulitis and colon micro-perforation following a Moderna booster dose in a young adult. Upon literature review performed on three databases - PubMed, Google Scholar, and ScienceDirect - this is the first published case report documenting adverse events profile post-COVID-19 vaccination.

## Case presentation

Our patient was a 41-year-old Caucasian male with a past medical history of bipolar depression, asthma, and obesity (body mass index: 40 kg/m2) who presented to the emergency room (ER) with the chief complaint of abdominal pain. The patient had received a Moderna COVID-19 vaccine booster dose the day prior to presentation to the ER. On the morning of the day of presentation to the ER, the patient started experiencing diarrhea and progressively worsening sharp, intermittent, generalized abdominal pain associated with sweating and loss of appetite. The patient rated intensity at 5 out of 10. The patient denied having any associated nausea/vomiting, fever, or chills. The patient had tolerated the first two doses of the vaccine without any adverse events. The patient’s surgical history included the presence of a stent in the internal carotid artery. No family history of cancer (including colon cancer), bleeding, or clotting disorders was reported. The patient was on aripiprazole and divalproex sodium at home for psychiatric issues. The patient had a history of vaping on a daily basis but no previous or current history of alcohol or substance abuse.

Vital signs on arrival to the ER showed a temperature of 97.8°F, heart rate of 95 bpm, respiration rate of 18 breaths/min, and blood pressure of 148/100 mm/Hg. The patient was maintaining 100% oxygen saturation on room air. On physical examination, the presence of generalized abdominal tenderness was appreciated on superficial and deep palpation, without any signs of rigidity, guarding, or peritonitis. The patient remained alert and oriented to time, place, and person, without any visible distress. Blood work including complete blood count (CBC) and basic metabolic panel (BMP) remained within the standard limits except for leukocytosis of 13.5 10x3/ul (granulocyte 71%, lymphocytes 17%), and are described in detail in Table 1.

**Table 1:** Blood work on day 0 (Admission) and day 2 (Discharge) WBC: White Blood Cell, Hb: Hemoglobin, MCV: Mean Corpuscular Volume, Na: Sodium, K: Potassium, AG: Anion Gap, S. creat: Serum Creatinine, GFR: Glomerular Filtration Rate, Ca: calcium, Mg: magnesium.

	Day 0	Day 2	Reference Range
WBC	13.5	7.1	4.0-11.0 10x3/ul
Hb	15.4	15.2	13.0-18.0 g/dL
MCV	87.5	88.0	80-95 fl
Platelet	251	333	150-450 10x3/uL
Na	135	137	136-145 mmol/L
K	3.9	4.3	3.5-5.1 mmol/L
Glucose	101	92	75-100 mg/dL
AG	07	05	8-16 mEQ/L
S. Creat	0.9	0.8	0.6-1.3 mg/dL
GFR	>60	>60	(>60 mL/min)
Ca	9.2	9.0	8.5-10.1 mg/dL
Mg	2.2	2.3	1.8-2.4 mg/dL

**Figure 1:**
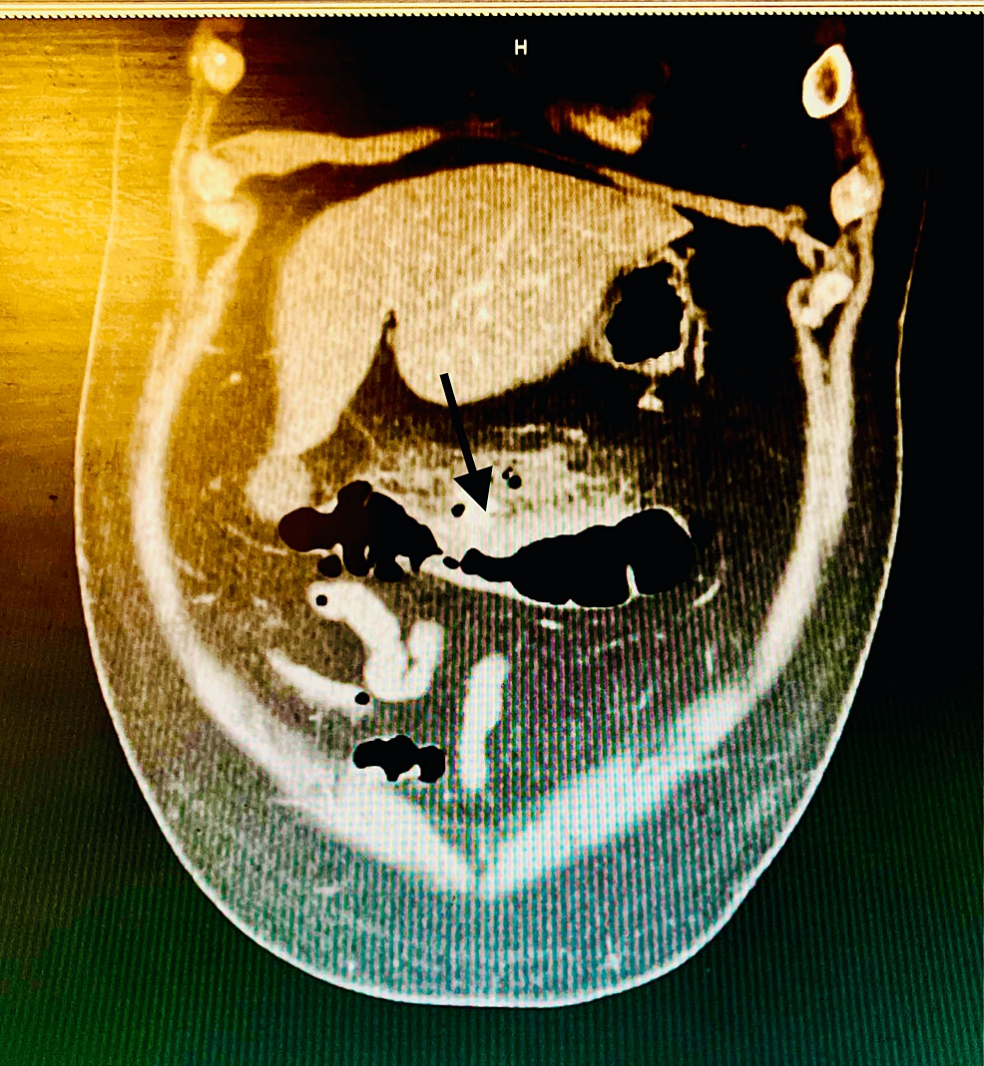
Diverticulitis transverse colon with extra-luminal air suggesting micro-perforation (arrow)

Urine drug screen remained negative for the presence of any opiates, methadone, barbiturates, benzodiazepines, phencyclidine, amphetamines, cocaine, or cannabinoids. SARS-CoV-2 (reverse transcription polymerase chain reaction - RTPCR) test results were negative. Computed tomography (CT) scan of the abdomen/pelvis without contrast showed acute diverticulitis of the mid-transverse colon, with adjacent contained micro-perforation. The patient was designated nil by mouth for bowel rest and started on empiric piperacillin/tazobactam antibiotic treatment along with intravenous hydration with normal saline for the diagnosis of acute diverticulitis, sepsis, and micro-perforation of the colon. On day 1, the patient remained afebrile and without visible signs of distress. White blood cell count improved to 10.0 × 103/μL. Hemoglobin remained stable at 14.7 g/dL. General surgery consult was obtained for further evaluation. Upon their recommendation, a CT scan of the abdomen with IV and oral contrast was obtained, which re-demonstrated stranding around the transverse colon, concerning for acute diverticulitis. Within this region, there remained a few areas of extraluminal air and fluid without a discrete drainable abscess, consistent with viscus perforation. However, imaging did not reveal any active leakage of the contrast, suggesting a sealed-off perforation, hence did not necessitate emergent surgical intervention (Figure 1).

On day 2, the patient’s abdominal pain improved significantly. The patient started to tolerate a clear liquid diet. Blood culture remained negative. Diet was advanced to mechanical, soft, and low-fiber, and the patient was discharged home on augmentin to complete a 10-day antibiotic course with stable complete blood count (CBC) and basic metabolic panel (BMP) at discharge (Table 1). The patient was recommended for outpatient follow-up with Gastroenterology, and to consider colonoscopy within 6 weeks.

## Discussion

Since the beginning of the COVID-19 pandemic, numerous treatment approaches (therapeutic and preventative) have been studied and implemented globally. While some measures have succeeded in controlling the pandemic, others have not. It is essential to recognize that to date there is no definite cure for COVID-19. Primary prevention of the disease via mass vaccination remains the main stem of the preventative approaches. In the US, 65% of the population has fully been vaccinated, and 76% has received at least one dose. Additionally, 44% of the US population has received a booster dose of the vaccine as of March 2022 [10]. Most adverse events caused by COVID-19 vaccines are transient and non-life-threatening and include pain, redness and swelling at the injection site, headaches, myalgia, fever, and nausea. Personal history of severe allergies/anaphylaxis is the only absolute contraindication to vaccination.

It is well-known that COVID-19 is not confined to the respiratory system and impacts most organ systems. The most commonly documented gastrointestinal (GI) tract symptoms include nausea, vomiting, diarrhea, loss of appetite, and abdominal pain [11,12]. Interaction between the viral spike protein and the host angiotensin-converting enzyme type 2 (ACE2) cell receptor explains such diverse symptoms. ACE2 cell receptors are found in the lungs and in abundance in the esophagus, ileum, and colon [13]. SARS-CoV-2 viral spike protein has a greater affinity for the human ACE2. This enables the virus to enter the GI tract, where it promotes inflammation. The mRNA vaccine carries messenger RNA (genetic information), which, once injected, gets translated into viral spike protein in the host. Our hypothesis is that transcribed viral spike protein binds to cells in the GI tract in a manner similar to that of the actual virus, promoting dysbiosis and inflammation. Hence, the vaccine is able to produce various GI symptoms in recipients, similar to patients infected with SARS-CoV-2. However, this hypothesis needs to be further explored in clinical trials.

Most of the diverticulitis cases are simple and can be managed on an outpatient basis. Transverse colonic diverticula (TCD) represent only 6% of all gastrointestinal tract diverticula and are usually detected as asymptomatic findings on endoscopic examination [14]. Patients with TCD are 15-20 years younger than patients with left-sided diverticulitis [15]. Bowel perforation is a well-known complication of acute diverticulitis that occurs secondary to severe inflammation as probably happened in our patient. For perforation alone, the 1-year mortality is 19% [16]. An individualized approach needs to be considered when proceeding with the surgery (i.e. colectomy) for the treatment of perforation or micro-perforation post diverticulitis. Performing colonoscopy 6 weeks post diverticulitis episode is the standard of care currently. Some of the other complications associated with diverticulitis are phlegmon, abscess, ascending septic thrombophlebitis (phylephlebitis), bleeding, intestinal obstruction, and fistula. While well-contained perforations usually are small and self-limiting, non-contained perforations may cause local abscess and fistula formation [17].

The Vaccine Adverse Events Reporting System (VAERS) is co-managed by the US CDC and the US Food and Drug Administration (FDA). Anyone can report an adverse event related to the vaccine to VAERS. Healthcare professionals are even required to report certain adverse events, and vaccine manufacturers must report all adverse events that come to their attention to VAERS. Even though the VAERS database is the only way to learn about vaccine-related adverse events, the data set has limitations, such as passive data collection and underreporting. Therefore, real-world data might reveal higher incidences of adverse events. Additionally, it is essential to keep in mind that VAERS data only establishes a temporal association, not a causal association. There is always a chance that some of the adverse events listed might be coincidental. Table 2 summarizes the GI tract adverse event profile of the three major COVID-19 vaccines administered (post first, second, and/or booster doses) in the US [18]. It is quite evident from these data that diarrhea and abdominal pain are the most common adverse events post-vaccination.

**Table 2:** GI adverse events post-covid-19 vaccination GI: gastrointestinal

Vaccine	Sex	Bowel Perforation	Diverticulitis	Diarrhea	Abdominal pain	GI Bleeding
JANSSEN	Female	04	32	1550	719	11
	Male	01	12	676	346	08
	Unknown	00	01	67	19	01
	Total	05	45	2293	1084	20
MODERNA	Female	10	179	8372	3128	34
	Male	06	77	2787	1273	31
	Unknown	00	01	224	109	00
	Total	16	257	11383	4510	65
PFIZER-BioNTech	Female	8	217	9546	3448	56
	Male	07	81	3392	1520	27
	Unknown	00	06	208	63	03
	Total	15	304	13146	5031	86
Total		36	606	26822	10625	171

Of 36 reported cases of bowel perforation post-vaccination, 13 patients have died (nine female, four male), and of 606 reported cases of diverticulitis post-vaccination, 15 individuals (eight female, seven male) have died [18]. Additionally, at least 100 vaccine recipients have died from GI bleeding among a total of 171 incidents post-vaccination, and 431 vaccine recipients reporting post-vaccination diarrhea (210 female, 221 male) have died among 26,822 total reported incidents of diarrhea post-vaccination, according to VAERS as of March 5, 2022 [18]. It is worth mentioning that >250 million people have been vaccinated with at least one dose [10]. Thus, the incidence of these adverse events can be considered a non-serious and rare adverse event, per the World Health Organization’s definition [19].

The female sex is associated with a higher overall incidence of all adverse events than the male sex. Furthermore, analyses of VAERS data suggest the 50-59-year age group for the female sex is associated with the highest incidence of adverse events for all three major vaccines. Such an association was not found with the male sex.

COVID-19 vaccines are safe in general and highly effective in reducing the incidence rate, mortality, and morbidity rate of the disease. However, vaccine recipients should be properly educated regarding GI adverse events post-vaccination. In case of an adverse event, the recipient should obtain immediate proper medical attention in order to prevent further complications and possible death.

## Conclusions

GI symptoms are not uncommon in patients with COVID-19, with the most common being diarrhea, followed by abdominal pain, nausea, vomiting, GI bleeding, and worsening of pre-existing GI conditions. Vaccines play a vital role in reducing overall morbidity and mortality from the disease. The benefits of the vaccines certainly outweigh the risks of adverse events. With time, the incidence of COVID-19 vaccine-related adverse events is becoming clearer. Most of the reported GI tract-related adverse events post-COVID-19 vaccination are non-life-threatening and transient. COVID-19 vaccine recipients should be properly educated on the complete adverse event profile of the vaccines. We also recommend vaccine recipients with pre-existing GI tract diseases, including those with a history of GI bleeding, inflammatory bowel disease (IBD), or peptic ulcer disease should be warned of the potential GI tract-related adverse events and closely monitored. Appropriate hydration should be promoted post-vaccination in addition to replacing electrolytes (with oral rehydration solution) in cases of intractable diarrhea, or when vaccine recipients experience GI bleeding, prompt medical attention should be sought to prevent hemorrhagic shock and reduce mortality/morbidity.
